# Is neoadjuvant chemoradiotherapy for pancreatic cancer beneficial: A systematic review and meta-analysis

**DOI:** 10.3389/fonc.2022.979390

**Published:** 2022-11-23

**Authors:** Wenhao Luo, Yawen Wang, Yinjie Tao, Taiping Zhang

**Affiliations:** ^1^ Department of General Surgery, Peking Union Medical College Hospital, Chinese Academy of Medical Sciences and Peking Union Medical College, Beijing, China; ^2^ Peking Union Medical College Hospital, Chinese Academy of Medical Sciences and Peking Union Medical College, Beijing, China

**Keywords:** neoadjuvant CRT, upfront surgery, overall survival, adverse events, complication events

## Abstract

To examine the potential benefits and adverse events of neoadjuvant Chemoradiotherapy (CRT) versus upfront surgery in pancreatic cancer (PC) patients. Extensive librarian-led literature searches were conducted on PubMed, Web-of-Science, Scopus, Google Scholar, the Cochrane Central Library and Embase. The primary outcomes were resectability, adverse events, pathological and survival outcomes. Five studies, including 437 participants, were analyzed. Upfront surgery had a significantly higher resectability among PC patients than neoadjuvant CRT group (Odds ratio = -0.11, 95% CI = -0.19–0.02, P = 0.01). The neoadjuvant CRT group had a comparatively higher Ro resection rate (OR = 3.38, 95% CI = 2.03–5.62, P < 0.01), fewer severe adverse events(OR = 0.56, 95% CI = 0.34–0.92, P = 0.02), lower positive LN rate(OR = 0.18, 95% CI = 0.11-0.31, P < 0.01) and higher 2-year OS(OR = 1.60, 95% CI = 1.02-2.52, P = 0.04) among PC patients than control group. There was no significant difference between neoadjuvant CRT and upfront surgery among PC patients on postoperative complications(OR = 1.49, 95% CI = 0.86-2.57, P = 0.16), metastasis rate(OR = 1.32, 95% CI = 0.42-4.18, P = 0.64) and 1-year OS(OR = 1.30, 95% CI = 0.85-1.98, P = 0.22). This systematic review confirmed the status of neoadjuvant CRT in the PC treatment. The neoadjuvant CRT could increase the R0 resection rate, which was important to the survival and life quality of patients. The specific choice of various neoadjuvant CRT therapy needs to be further studied. Individualized neoadjuvant therapy should be suitable for each patient, and patients with PC are best managed by a multidisciplinary team.

## Introduction

Despite the continuous improvement in the diagnosis and treatment of pancreatic cancer (PC), the mortality of PC is still increasing, with a five-year survival rate of only 10% (1). New treatment for PC is still in urgent need of exploration. Surgery is the primary treatment for PC at present. The prognostic factors of PC include tumor size, lymph node metastasis, histological grade and adjuvant therapy (2). Adjuvant therapy for PC has achieved significant efficacy in patients after surgery. However, the two-year recurrence rate remains high, and it is hard for patients with postoperative complications to tolerate adjuvant therapy (3).

The neoadjuvant therapy is to apply chemotherapy and/or radiotherapy before surgery (4). With the significant progress of neoadjuvant therapy in digestive tract tumors, such as rectal cancer, gastric cancer, and esophageal cancer, the effect of neoadjuvant therapy on pancreatic cancer has been explored in many studies (5). The neoadjuvant therapy could reduce the scope of lesions and improve the rate of complete tumor resection. Meanwhile, patients receiving neoadjuvant therapy are not affected by surgical complications (4). The disadvantage is that neoadjuvant therapy may delay the timing of surgery for PC (4).

Whether neoadjuvant therapy can achieve better survival benefits than up-front surgical treatment is still controversial (5). In a multi-institutional phase II trial reported by Talamonti, preoperative full-dose gemcitabine and radiotherapy were applied to patients with potentially resectable pancreatic cancer, reducing the margin and node involvement (6). Kim et al. reported a multi-institutional phase 2 study, showing that full-dose gemcitabine, oxaliplatin, and radiation therapy could increase the rate of R0 resections (7). Recently, many studies and larger cohorts were published to compare neoadjuvant therapy with up-front surgical treatment to prove its safety and effectiveness (8, 9). However, no consistent conclusion was reached in various outcomes, such as overall survival (OS), adverse events and efficacy. A systematic review and meta-analysis is needed to find the comprehensive effect.

This article searched the randomized controlled trials (RCT) comparing neoadjuvant chemoradiotherapy (CRT) and up-front surgical therapy for PC. We discussed the differences in resectability, Ro resection rate, positive lymph nodes rate, severe adverse events, metastasis rate and overall survival (OS). This article aims to provide a potential direction for the treatment of PC and further improve the survival benefit of patients.

## Methods

This systematic review and meta-analysis was completed according to the Preferred Reporting Items for Systematic Reviews and Meta-analysis (PRISMA) statement (10).

### Search selections

Relevant studies from the extensive librarian-led literature search of PubMed, Web-of-Science, Scopus, Google Scholar, the Cochrane Central Library and Embase were downloaded on 15 May 2022. In addition, a manual search was completed to avoid missing relevant articles. The search strategy included the medical subject headings (MESH) or the following terms: “neoadjuvant”, “chemoradiotherapy or radiochemotherapy”, “pancreas/pancreatic”, “cancer/carcinoma/adenocarcinoma”, “randomized/randomized controlled study” and “Human”. Only English articles and randomized controlled trials (RCTs) published in full peer-reviewed journals were included strictly. PC Patients who applied neoadjuvant CRT treatment were included. Non-comparative studies were excluded from this meta-analysis.

### Data extraction

Included studies were independently reviewed by two authors (LWH and TYJ). The following terms were extracted, including baseline characteristics and outcome information: the first author, published year of study, type of treatment, number of participants and all the relevant outcomes. The outcome measure included (1) Resectability; (2) Ro resection rate; (3) Positive lymph nodes rate; (4) severe adverse events; (5) postoperative complications rate;(6) metastasis rate (7) 1-year OS; and (8) 2-year OS. We crosschecked to rule out the discrepancies. Disagreements were addressed through discussion until consensus was achieved.

### Estimation of evidence quality

The Critical Appraisal Skills Programme (CASP) Checklist was applied to evaluate the quality of evidence by two independent authors. A group discussion was completed to check the significant difference between the scores of the two authors. CASP Checklists assess the bias risk and comprise 11 items for evaluation (**Supplementary Table 1**). A minimal score of 0 for the total score means the lowest quality, while a maximum score of 11 represents the highest quality RCTs. Funnel plot figures were made to evaluate the publication bias (11).

### Statistical method

Meta-analyses were performed using the latest version of Reviewer Manager software (RevMan version 5.4; Cochrane Collaboration, Oxford, UK). For dichotomous outcomes, we analyzed the odds ratio (OR) with a 95% confidence interval (CI). Fixed-effects or random-effects models were used to combine the summary data accordingly. Tests of heterogeneity (*I* ^2^ index) were assessed by the chi test to evaluate the inconsistency between RCTs. We regarded I^2^ scores of 0–39% as unimportant, 40–60% as moderate, 60–75% as substantial and >75% as considerable heterogeneity. Publication bias was assessed by funnel plot figures. All statistical tests were performed at 5% significance level.

## Results

### Characteristics of included studies

**Figure 1:**
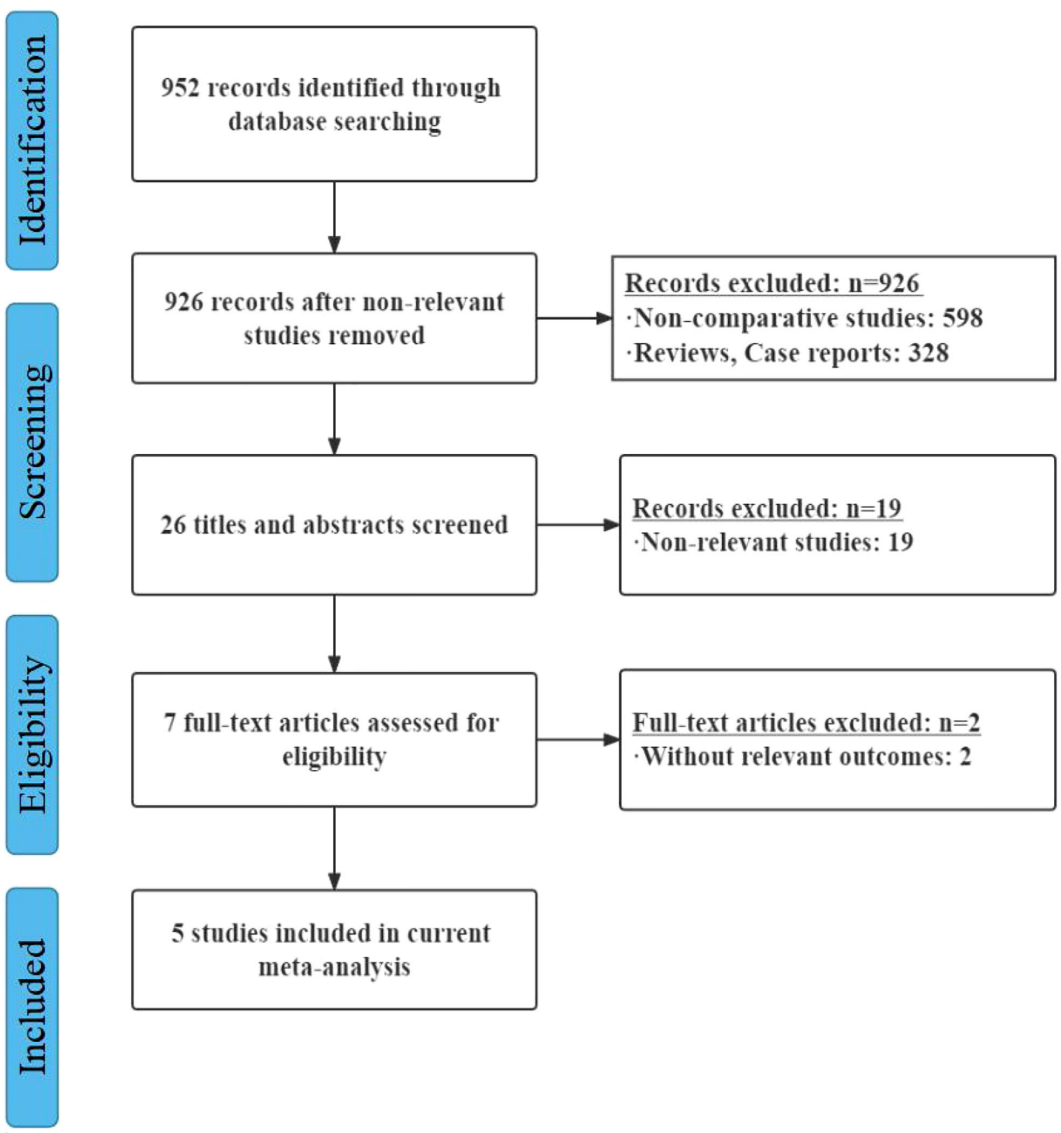
Flow diagram of the bibliographic search.

5 studies were eventually included and analyzed in the systematic review, accounting for 437 patients (8, 9, 12–14). We drew a flow-process diagram to show the whole process of our search (**Figure 1**). First, we identified 952 potentially eligible articles from the database searches. Non-RCT studies or not English articles were excluded. Then 26 articles were assessed by a careful reading of the abstracts. After thorough and detailed insights into these 26 full-text articles. 19 studies were excluded because CRT and upfront surgery were not compared. 2 studies were further excluded due to the lack of relevant outcomes. 5 studies were eventually included.

### Study characteristics

**Table 1** describes the basic information and characteristics of the included studies. Our systematic review and meta-analysis included 437 participants. Among them, 215 patients were treated with CRT, and 222 patients were treated with upfront surgery. The quality evaluation of all included trials is demonstrated in **Table 2**.

**Table 1:** Baseline information of randomized controlled trials enrolled in the meta-analysis.

Reference	Number of Patients, N		Classification of Tumor	Treatment arrangement in control group	CRT group			AT regimen in both groups	Treatment interval before surgery	
	CRT group	Control group			Treatment arrangement in CRT group	RT regimen in CRT group	Concurrent CT regimen in CRT group		CRT group	Control group
Golcher et al., 2015 (9)	33	33	resectable PC	surgery+AT	CRT+surgery+AT	3DCRT, 50.4Gy	300 mg/m^2^ Gemcitabine, 30 mg/m^2^ Cisplatin D1,8,22,29	Gemcitabine	11.5 weeks	upfront surgery
Joo et al., 2017 (13)	18	19	BR PC	surgery	CRT+surgery	3DCRT, 45Gy/18 fraction+9Gy/5 fraction boosting	Neoadjuvant chemoradiation; 45Gy/25fx + 9Gy/5fx of radiation over 6 weeks with 400mg/m^2^	within 4~6 weeks after operation with Gemcitabine 1000mg/m^2^ (D1, 8, 15) every 4 weeks, for 4 cycle	10 weeks	upfront surgery
Versteijne et al., 2020 (14)	119	127	resectable PC 133 BR PC 113	surgery+AT	CRT+AT	36Gy/15 fraction	1000 mg/m2 Gemcitabine, D1,8,15, 4 weeks	1000 mg/m2 Gemcitabine D1,8,15, 4 weeks	14-18 weeks	upfront surgery
Jang et al., 2018 (12)	27	23	BR PC	surgery+AT	CRT+AT	3DCRT, 45Gy/25 fraction+9Gy/5 fraction boosting	400 mg/m^2^ Gemcitabine	1000 mg/m2 Gemcitabine D1,8,15, every 4 weeks for 4 cycles	Ns	upfront surgery
Casadei et al., 2015 (8)	18	20	resectable PC	surgery+AT	CRT+AT	45Gy, boost of 9Gy	50 mg/m^2^ Gemcitabine	Gemcitabine	16-18 weeks	upfront surgery

*BR PC, borderline resectable pancreatic cancer; LA PC, locally advanced pancreatic cancer; AT, adjuvant chemotherapy; IC, induction chemotherapy; CRT, chemoradiotherapy; 3DCRT, 3D conformal radiotherapy. Ns, Not specific.

**Table 2:** Quality evaluations of RCTs finally included in the meta-analysis.

Reference	Score of item I	Score of item II	Score of item III	Score of item IV	Score of item V	Score of item VI	Score of item VII	Score of item VIII	Score of item IX	Score of item X	Score of item XI	Total scores
Golcher (9)	1	1	0.5	1	0	0	1	1	1	1	1	8.5
Joo 2017 (13)	1	1	0.5	1	0.5	0	1	1	1	1	1	9
Versteijne (14)	1	1	0.5	1	0	0	1	1	1	1	1	8.5
Jang (12)	1	1	1	1	0	0	1	1	1	1	1	9
Casadei (8)	1	1	0.5	1	0	0	1	1	1	1	1	8.5

### Primary outcomes

### Resectability

**Figure 2:**
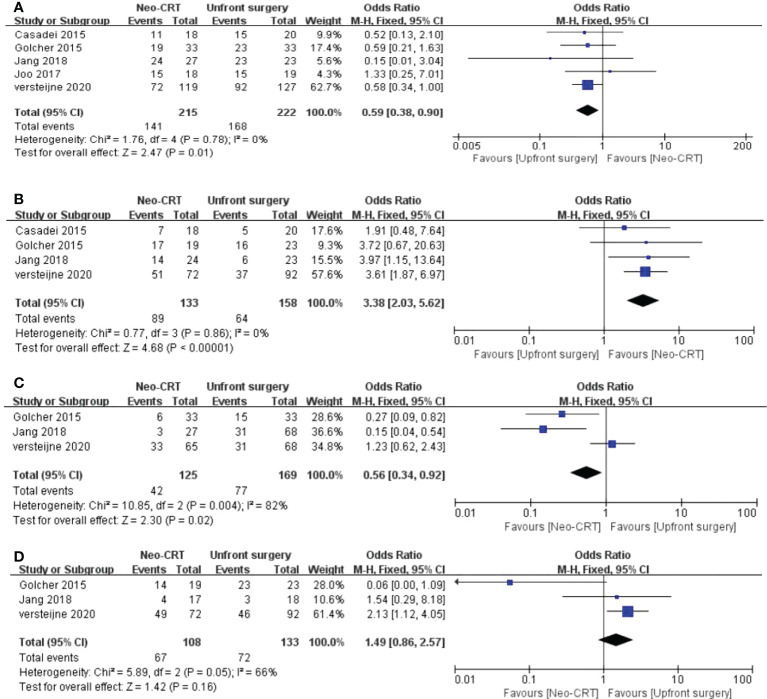
Meta-analysis of study on resectability and adverse events outcomes. Forest plot of **(A)** Resectability; **(B)** R0 rate; **(C)** Severe adverse events; **(D)** postoperative complications.

Five articles reported resectability. All five articles showed that upfront surgery has a significantly higher resectability among PC patients than the neoadjuvant CRT group. (Odds ratio = 0.59, 95% CI = 0.38–0.90, P = 0.01) (**Figure 2A**).

### Ro resection rate

Four articles reported the Ro resection rate evaluation between the neoadjuvant CRT group and the upfront group. We found that the neoadjuvant CRT group has a comparatively higher Ro resection rate among PC patients than the control group. (OR = 3.38, 95% CI = 2.03–5.62, P < 0.01, I^2 =^ 0%) (**Figure 2B**).

### Severe adverse events

Three articles compared the severe adverse events between neoadjuvant CRT and the upfront surgery group. The neoadjuvant CRT has fewer severe adverse events than the upfront surgery group among PC patients. (OR = 0.56, 95% CI = 0.34–0.92, P = 0.02, I^2 =^ 82%) (**Figure 2C**).

### Post-operative complications rate

Three articles reported the complication rate. There is no significant difference between neoadjuvant CRT and upfront surgery among PC patients in postoperative complications.(OR = 1.49, 95% CI = 0.86-2.57, P = 0.16, I^2 =^ 66%) (**Figure 2D**).

### Positive lymph nodes rate

**Figure 3:**
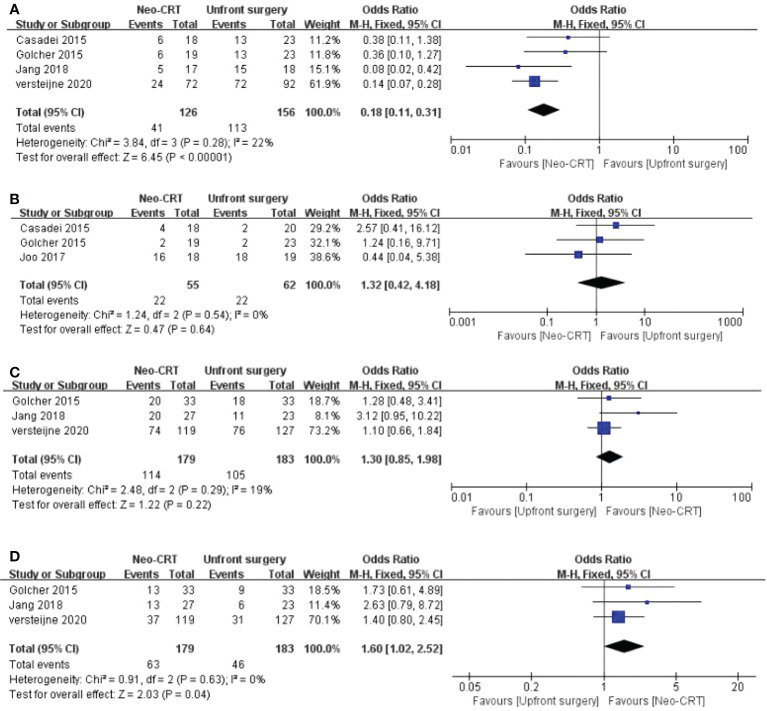
Meta-analysis of study on pathological and survival outcomes. Forest plot of **(A)** positive lymph node rate; **(B)** metastasis rate; **(C)** 1-year OS; **(D)** 2-year OS.

Four articles reported the Positive lymph nodes rate. Neoadjuvant CRT has a comparatively lower positive LN rate than the control group among PC patients. (OR = 0.18, 95% CI = 0.11-0.31, P < 0.01,I^2 =^ 22%) (**Figure 3A**).

### Metastasis rate

Three articles reported the metastasis rate. There is no significant difference between neoadjuvant CRT and upfront surgery among PC patients on metastasis rate. (OR = 1.32, 95% CI = 0.42-4.18, P = 0.64,I^2 =^ 0%) (**Figure 3B**).

### 1-year OS

Three articles reported the 1-year OS. There is no significant difference between neoadjuvant CRT and upfront surgery among PC patients on 1-year OS. (OR = 1.30, 95% CI = 0.85-1.98, P = 0.22,I^2 =^ 19%) (**Figure 3C**).

### 2-year OS

Three articles reported the 2-year OS. The neoadjuvant CRT has a comparatively higher 2-year OS than the control group among PC patients. (OR = 1.60, 95% CI = 1.02-2.52, P = 0.04,I^2 =^ 0%) (**Figure 3D**).

### Potential publication bias

**Figure 4:**
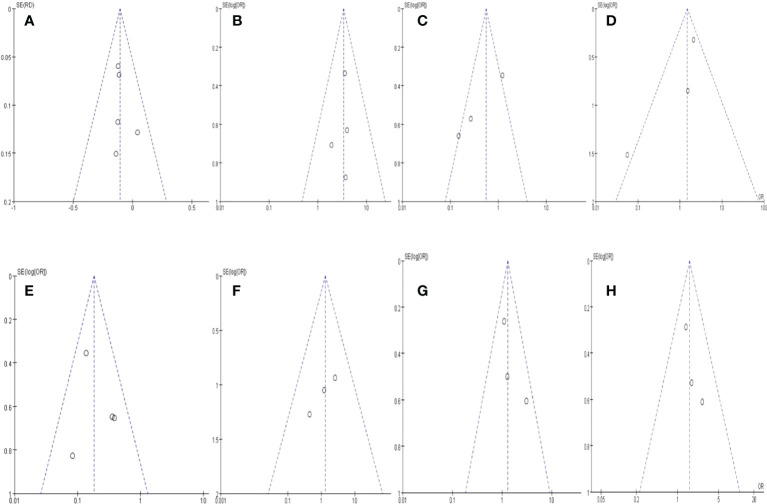
Funnel plot of outcomes.s. **(A)** Resectability; **(B)** R0 rate; **(C)** Severe adverse events; **(D)** postoperative complications, **(E)** positive lymph node rate; **(F)** metastasis rate; **(G)** 1-year OS; **(H)** 2-year OS.

A funnel plot regarding (a) Resectability; (b) R0 rate; (c)Severe adverse events; (d) postoperative complications, (e) positive lymph node rate; (f) metastasis rate; (g) 1-year OS; (h) 2-year OS. are demonstrated in **Figure 4**, respectively. No apparent asymmetry was shown through the funnel plot, and only 1 study exceeded the 95% CI for postoperative complications. There was no funnel plot asymmetry, suggesting no significant publication bias among all evaluated outcomes of those included RCTs.

## Discussion

This is the first systematic review and meta-analysis of all relevant RCTs to show the advantages of neoadjuvant CRT therapy in PC. As we know, surgical resection has been considered the only curative treatment for pancreatic cancer. Due to the high margin-positive rate and positive LN rate, even extended resection cannot prove a higher overall survival rate (15). Therefore, it is of great significance to adopt a different treatment method for PC with higher R0 resection rates, OS, curative resection and lower invasive status. With the development of neoadjuvant therapy and the change of concept from’surgery first’ to ‘surgery last’, it is feasible to use moving chemoradiotherapy preoperatively.

In this meta-analysis, we identified a significant trend toward improved 2-year OS in the neoadjuvant CRT groups than the upfront surgery group. In the neoadjuvant CRT group, the R0 resection rate, and 2-year OS were higher, while the positive LN rate, severe adverse events were lower than upfront surgery. There was no difference in postoperative complications, metastasis rate, and 1-year OS between neoadjuvant CRT and upfront surgery among PC patients. These findings support that neoadjuvant CRT is an appropriate treatment for PC patients with reliable efficacy and safety. The improved survival rate of neoadjuvant CRT probably results from the increased R0 resection, decreased positive LN rate and lower severe adverse events. In those included RCTs, we found that all neoadjuvant CRTs are based on gemcitabine, suggesting gemcitabine-based neoadjuvant CRTs are widely explored.

Generally, tumor removal could be categorized into R_0_、R_1mm_、R_2_ removal according to the pathological examination. Notably, R_0_ resectability was the only key to improving long-term overall survival (16). In patients who accepted upfront surgery, more patients had the opportunity of tumor removal, but the R_0_ resectability rate was lower. It suggested that a significant proportion of patients underwent R_1_ or R_2_ surgical removal in the control group. For those patients, the clinical benefits of surgery were unclear. Previous studies have proven that margin-positive pancreaticoduodenectomy would not improve survival or life quality compared with palliative bypass surgical treatment (17). Conversely, pancreatic cancer cells are heterogeneous in potential invasiveness and progression. Upfront surgery could result in 30% early recurrence or metastasis due to invisible spreading spots in retroperitoneal tissues or nervous tissues. Neoadjuvant chemoradiotherapy provides a window to observe the tumor’s biological behavior and to screen patients who cannot benefit from upfront surgery.

There are several explanations for how neoadjuvant CRT might have improved oncologic outcomes. First, although PC responsiveness to neoadjuvant CRT varied greatly, the neoadjuvant CRT may reduce the tumor mass, limit the undetected micro-metastasis, and control the tumor aggressiveness at risk for early recurrence through gemcitabine, leading to higher R0 resection rate and better prognosis. Evans et al. demonstrated that initially unknown micro-metastases might be eliminated by preoperative therapy (18). Moreover, a recent study showed that CRT with full-dose gemcitabine may have local and systemic effects in improving the R0 resection rate and reducing micrometastases (19). In this meta-analysis, only 24 out of 72 total patients (30.5%) in the neoadjuvant CRT group had positive LN compared to 72 out of 92 (78.3%) total patients with positive LN rate in the upfront surgery group. Moreover, 89 out of 133 total patients (66.9%) in the neoadjuvant CRT group had R0 resection compared to 64 out of 158 (40.5%) total patients with positive LN rate in the upfront surgery group, which is consistent with recent clinical trials. Considering that the surgery was standardized in both groups, neoadjuvant CRT treatment reduces the tumor burden of the primary tumor. A second explanation for improved outcomes is that neoadjuvant CRT had similar post-operative complications and lower severe adverse events than upfront surgery. Recent research demonstrated that gemcitabine-based pre-operative chemoradiotherapy is a safe and effective treatment of pancreatic ductal adenocarcinoma, which is consistent with our results. A third possible explanation is that neoadjuvant CRT induces a local downstaging effect on the tumor. Recent RCT showed that clinical tumor staging was down-staged after treatment of gemcitabine-based CRT (9). Another study showed that neoadjuvant CRT can downstage the PC and eventually increases margin-negative and node-negative rates at resection (20).

Upfront surgery is still many surgeons’ favorite choice for resectable or borderline resectable PC. Some patients may not show great responsiveness to neoadjuvant CRT and probably miss the best timing for curative resection. Conversely, many types of research proved the advantage of neoadjuvant treatment for resectable PC patients (21, 22), and the NCCN guidelines recommend neoadjuvant treatment for BRPC (23). Consequently, this meta-analysis demonstrated the benefit of neoadjuvant CRT over upfront surgery. It may provide novel insight and solid evidence for future investigation.

The studies on neoadjuvant treatment and the comparison of different NAT regimens are still limited. Chemotherapy regimens and modes of various radiotherapy techniques may lead to different outcomes. The modified FOLFIRINOX regimen has shown its importance in the neoadjuvant treatment of patients with PC (24). The AG regimen (Albumin paclitaxel and gemcitabine) was also prevalently used and relatively safe (25). The status of preoperative radiation is still under investigated. Several trials concluded that radiation was essential in the neoadjuvant regimen and potentially improved the prognosis (26, 27). The role of RT was recognized as an alternative to shrink tumor mass and reduce local recurrence. The safety of radiation was guaranteed, but there was no consensus on whether RT could improve survival. In the current study, we investigated the CRT regimen as a combination. The clinical outcomes of CT and CRT were not compared due to the limited number of prospective clinical trials. Higher-level evidence was needed in the following research. Besides, reliable methods to re-estimate the resectability of the tumor after NAT was absent. Currently, surgical exploration is recommended for all patients after receiving NAT if there is no significant disease progression (28).

The limitation of the meta-analysis is that only RCTs were included in the final meta-analysis. The highlight of the study was that we only included high-quality RCTs to ensure the reliability of the conclusions. Many comparative studies are comparing neoadjuvant CRT with upfront surgery. However, RCTs endowed characteristics with a prospective, comparative, quantitative research under controlled conditions with randomly assigned intervention measures to the control group (29). Only RCTs were included in this article to ensure high-quality evidence from a systematic review and meta-analysis. Moreover, it is unlikely to erase all the heterogeneity of various outcomes between the neoadjuvant CRT and the upfront surgery group. But only RCTs have been included, there is a balanced inconsistency between the two groups in the meta-analysis. The heterogeneity between each RCT, such as tumor operability, details of the neoadjuvant therapy and the selection of endpoints, was minimized but inevitable during the analysis. In addition, all included participants were resectable PC patients or borderline resectable PC patients. There are no unresectable PC patients in this analysis, because it is impossible to perform an upfront surgery for unresectable PC patients for randomized control. Hence, no relevant RCTs compare neoadjuvant CRT and upfront surgery for unresectable PC patients.

## Conclusion

This is the first systematic review and meta-analysis of all RCTs on preoperative CRT and immediate surgery in PC. This systematic review and meta-analysis found that neoadjuvant CRT significantly increases the R0 resection rate and the 2-year OS, decreases the severe adverse events and positive LN rate compared with upfront surgery. Therefore, neoadjuvant CRT is a recommendable treatment for patients with resectable or borderline resectable PC. Although neoadjuvant CRT has better outcomes than upfront surgery, the improved short-term or long-term results for PC are still limited. Therefore, in future research, more clinical trials could explore and focus more on the novel or modified neoadjuvant CRT.

## Data availability statement

The original contributions presented in the study are included in the article/**Supplementary Material**. Further inquiries can be directed to the corresponding author.

## Author contributions

Study design: WL; Literature search: WL, YT, and YW; Study selection: WL; Study draft and revision: WL; Article guarantor: TZ. All authors contributed to the article and approved the submitted version.

## Funding

This study was supported by grants from the National Key R&D Program of China (2018YFE0118600); National Multidisciplinary Cooperative Diagnosis and Treatment Capacity Building Project for Major Diseases; National Natural Science Foundation of China (No.81972258;81974376;82103016;82172836;82272917;82203158); CAMS Innovation Fund for Medical Sciences (CIFMS) (2021-1-I2M-002); China Postdoctoral Science Foundation (2021T140071 and 2021M690462); Youth Research Fund of Peking Union Medical College Hospital (pumch201911710, pumch201910819); National Multidisciplinary Cooperative Diagnosis and Treatment Capacity Building Project for Major Diseases. National High Level Hospital Clinical Research Funding (2022-PUMCH-A-056;2022-PUMCH-A-133;2022-PUMCH-A-245).

## Acknowledgments

We acknowledge all the participants in searching, analyzing and concluding those studies that contributed to this piece of work and all the collaborators who made such studies possible. We acknowledge the clinic staff and managers of Peking Union Medical College Hospital, Chinese Academy of Medical Sciences and Peking Union Medical College for their valuable contributions to this research. By the way, YW and YT contributed to this article equally.

## Conflict of interest

The authors declare that the research was conducted in the absence of any commercial or financial relationships that could be construed as a potential conflict of interest.

## Publisher’s note

All claims expressed in this article are solely those of the authors and do not necessarily represent those of their affiliated organizations, or those of the publisher, the editors and the reviewers. Any product that may be evaluated in this article, or claim that may be made by its manufacturer, is not guaranteed or endorsed by the publisher.
