# Supplementary material for: Is neoadjuvant chemoradiotherapy for pancreatic cancer beneficial: A systematic review and meta-analysis
Source: Front Oncol. 2022 Nov 23;12:979390. doi: 10.3389/fonc.2022.979390 (PMC9727153; doi:10.3389/fonc.2022.979390)
Supplement: Supplementary file 1 [file Table_1.docx]

Supplementary Table 1 Critical Appraisal Skills Programme (CASP) Checklist

| Item Number | Items of quality assessment |
| --- | --- |
| 1 | Was the assigned treatment adequately concealed before allocation? |
| 2 | Were the outcome of patients who withdrew described and included in the analysis (intention to treat)? |
| 3 | Were the outcome assessors blinded to the treatment status? |
| 4 | Were the treatment and control groups comparable at entry? |
| 5 | Were the participants blinded to the assignment status after allocation? |
| 6 | Were the treatment providers blind to the assignment status? |
| 7 | Were the care programs, other than the trial options, identical? |
| 8 | Were the inclusion and exclusion criteria clearly defined? |
| 9 | Were the interventions clearly defined? |
| 10 | Were the outcome measures used clearly defined? |
| 11 | Were diagnostic tests used in the outcome assessment clinically useful? |
